# Retrospective Analysis of Oral Tofacitinib Treatment for Alopecia Areata at a Tertiary Care Hospital in Saudi Arabia

**DOI:** 10.7759/cureus.101063

**Published:** 2026-01-08

**Authors:** Faisal A Aleidi, Monirah O Almadhi, Ziyad H Altassan, Lama F Alotaibi, Bijad Almutairi, Mona Khalaf, Haneen M Almansour, Raghad R Alhowil, Ahmed Alotaibi, Sadeem A Alsultani, Raghad Fahad, Fay Thawab

**Affiliations:** 1 Pharmacy Service Administration, King Fahad Medical City, Riyadh, SAU; 2 College of Pharmacy, Shaqra University, Shaqra, SAU; 3 College of Pharmacy, Qassim University, Buraydah, SAU; 4 College of Pharmacy, King Saud Bin Abdulaziz University for Health Sciences, Riyadh, SAU

**Keywords:** alopecia areata, janus kinase inhibitors, real-world, salt score, tofacitinib

## Abstract

Background: Alopecia areata (AA) is a chronic autoimmune disorder characterized by non-scarring hair loss and substantial psychological burden. Although Janus kinase (JAK) inhibitors such as tofacitinib have shown promising efficacy, evidence on their long-term real-world use, particularly in Middle Eastern populations, remains scarce. This study addresses this gap by evaluating extended real-world outcomes of oral tofacitinib in a Saudi Arabian cohort.

Methods: This retrospective cohort study evaluated patients with AA treated with oral tofacitinib at a tertiary care center between 2018 and 2024, providing up to six years of follow-up. Patients who received tofacitinib for at least 12 months were included. Demographic data, disease characteristics, treatment regimens, clinical response, adverse events, and laboratory parameters were extracted from electronic medical records. Associations were analyzed using appropriate statistical tests.

Results: Sixteen patients were included, 56.3% female (n = 9), with a mean age of 29.6 ± 9.2 years. Most patients had AA (93.8%, n = 15), and one had alopecia totalis (6.3%, n = 1). An effective response was achieved in 68.8% of patients (n = 11), while 31.3% (n = 5) showed no meaningful response. Hyperlipidemia was the most frequent adverse event (87.5%, n = 14) and was managed conservatively or with lipid-lowering therapy without treatment discontinuation. No serious adverse events or cardiovascular complications were observed. Ongoing side effects (62.5%, n = 10) were significantly associated with treatment response (p = 0.018). Some patients required dose escalation or were switched to alternative JAK inhibitors with favorable outcomes.

Conclusion: In this six-year real-world Saudi cohort, oral tofacitinib demonstrated favorable long-term effectiveness in patients with AA. Despite frequent lipid abnormalities, adverse events were manageable with routine monitoring, supporting the feasibility of long-term tofacitinib therapy in carefully selected patients.

## Introduction

Alopecia areata (AA) is a common autoimmune disorder that causes non-scarring hair loss on the scalp and other body areas. It occurs when the immune system mistakenly attacks hair follicles, leading to patchy or total hair loss. The condition can affect individuals of any age and often follows an unpredictable course of relapse and regrowth, causing significant emotional and psychological distress for many patients [1]. The prevalence of AA in Saudi Arabia was found to be between 2.3% and 5.2% in recent studies, a prevalence higher than that in Western countries. Patients in the Saudi population were also observed to present at younger ages [2].

AA significantly impairs quality of life due to its visible and unpredictable nature, adversely affecting self-image, self-esteem, and social interactions. Hair loss often leads to emotional distress and social withdrawal. Studies have shown that individuals with AA experience higher rates of anxiety and depression compared with the general population. The chronic and relapsing course of the disease further intensifies psychological burden, underscoring the importance of addressing psychosocial aspects in comprehensive patient care [3]. Tofacitinib, a Janus kinase (JAK) 1 and 3 inhibitor, is one of the earliest JAK inhibitors investigated for the treatment of AA. It acts by blocking the JAK-STAT signaling pathway, which mediates interferon-γ and other pro-inflammatory cytokines implicated in the autoimmune destruction of hair follicles. This mechanism provides a strong biological rationale for its use as a disease-modifying therapy rather than symptomatic immunosuppression [4]. Early clinical and real-world data have demonstrated that tofacitinib can induce substantial hair regrowth, even in patients with severe or treatment-resistant AA.

A landmark retrospective study by Ibrahim et al. showed that 54% of patients achieved ≥50% improvement in the Severity of Alopecia Tool (SALT) score after oral tofacitinib 5 mg twice daily, with acceptable tolerability [5]. Subsequent meta-analyses have confirmed meaningful response rates, reporting pooled good or complete regrowth in approximately 50-60% of treated patients [6]. Although tofacitinib is not formally approved for AA, it is increasingly prescribed off-label based on its JAK-inhibition mechanism and encouraging outcomes from real-world cohorts. Off-label use underscores the need for robust data on effectiveness, durability, safety, and relapse, along with careful patient selection, informed consent, and ongoing monitoring. Recent real-world studies reinforce its benefit: In a Chinese cohort study (n = 125), the treatment duration was three months, with an overall response rate of 83% and complete remission achieved in 16% of patients at three months, without any serious adverse events [7], while a U.S. retrospective cohort (Massachusetts General Hospital, n = 30) demonstrated 66.7% complete or near-complete regrowth over a median of 18.6 months, with only mild, reversible adverse effects such as transient lipid elevation or rash [8]. Several long-term studies have evaluated tofacitinib for AA. Husein-ElAhmed et al. followed 47 Arab-Asian patients (2018-2020) and reported 42% complete and 26% partial regrowth, recommending therapy for approximately 12 months to prevent relapse. A retrospective study of 126 patients (2021-2022) found a median treatment duration of 23 weeks, with 33.8% achieving complete response at 24 weeks and drug survival rates of 90%, 66%, and 42% at 12, 24, and 48 weeks, respectively. Notably, Al Ali et al. conducted the longest real-world follow-up study in the Middle East, including 45 patients treated with tofacitinib for 12 to 72 months (average 30 months), where 78% achieved moderate-to-significant hair regrowth. The treatment was well tolerated, and hair loss after stopping therapy typically reversed upon resuming treatment [9-11].

In light of the limited real-world evidence and the absence of long-term data on tofacitinib use for AA in Saudi Arabia, the present study contributes by documenting six years’ clinical outcomes in 16 patients, thereby filling an important gap in the regional literature.

## Materials and methods

This retrospective cohort study was conducted at King Fahad Medical City (KFMC), Riyadh, Saudi Arabia, using electronic medical records of patients diagnosed with AA who were treated with oral tofacitinib between January 2018 and December 2024. The primary objective of this study was to evaluate the long-term real-world effectiveness of oral tofacitinib in Saudi patients with AA. Secondary objectives included assessment of treatment safety, laboratory changes, treatment modifications, and clinical factors associated with treatment response over a six-year follow-up period.

Patients were eligible for inclusion if they had a confirmed diagnosis of AA based on documented clinical and dermoscopic findings, received oral tofacitinib for a minimum duration of 12 months, and had complete baseline and follow-up clinical data available for review, including disease severity assessment. Patients were excluded if they were treated with tofacitinib for indications other than AA, had diffuse alopecia not consistent with AA, or had incomplete follow-up or missing key clinical or laboratory data. Demographic and clinical variables were extracted from the EPIC electronic medical record system (Epic Systems Corporation, Verona, Wisconsin, USA) using a standardized data collection form. Collected data included age, sex, body weight, AA subtype, prior treatment history, tofacitinib dosing regimen and duration, treatment modifications, laboratory parameters, reported adverse events, and clinical response to therapy. Disease severity was assessed using the SALT score, a validated quantitative measure used to estimate the percentage of scalp hair loss. The SALT score is calculated by evaluating hair loss across four scalp regions-vertex (40%), right profile (18%), left profile (18%), and occipital region (24%) - with the weighted percentages summed to generate a total score ranging from 0 (no hair loss) to 100 (complete scalp hair loss) [12]. All patients underwent baseline laboratory investigations prior to initiating tofacitinib therapy, including complete blood count, liver and renal function tests, and lipid profile, with periodic monitoring throughout treatment.

The primary outcome was treatment effectiveness assessed using the SALT score. Treatment response was defined as the change in SALT score from baseline to the end of treatment. Patients achieving a clinically meaningful improvement, defined as a ≥50% reduction from baseline (SALT50) or a final SALT score ≤25, were classified as effective responders. All other patients, including those with minimal change, no improvement, or worsening, were classified as ineffective responders. Secondary outcomes included the incidence and type of adverse events, changes in laboratory parameters (particularly lipid profile), need for dose escalation or treatment switching, and identification of clinical factors associated with treatment response.

Categorical variables (e.g., gender, diagnosis, prior treatment, and treatment response) were summarized using frequencies and percentages, and group comparisons were performed using the chi-square test or Fisher’s exact test, as appropriate. Continuous variables (e.g., age, weight, treatment duration, and low-density lipoprotein (LDL) levels) were presented as means ± standard deviations and compared between groups using the independent samples t-test. All statistical tests were two-tailed, and a p-value < 0.05 was considered statistically significant. Analyses were conducted using IBM SPSS Statistics for Windows, Version 25 (Released 2017; IBM Corp., Armonk, New York, United States) and MedCalc version 23.0.2 (MedCalc Software Ltd., Ostend, Belgium). Ethical approval for the study was obtained from the Institutional Review Board of King Fahad Medical City (H-01-R-012), and all patient data were anonymized to ensure confidentiality in accordance with institutional and ethical guidelines.

## Results

A total of 16 patients were included in this analysis. Most patients were diagnosed with AA (93.8%), with only one case of alopecia totalis (6.3%). The study population consisted of 56.3% females and 43.8% males, with a mean age of 29.56 ± 9.21 years and an average body weight of 67.12 ± 17.91 kg. Prior to the current treatment, half of the patients (50.0%) had used topical minoxidil 5%, while 31.3% had received no previous therapy. The majority (75.0%) started treatment at a dose of 5 mg twice daily, and the mean treatment duration was 3.81 ± 1.27 years. In terms of therapeutic response, 68.8% of patients achieved an effective response, whereas 31.3% showed no meaningful improvement. Side effects were frequent, with hyperlipidemia occurring in 87.5% of patients, and one patient additionally reported a headache. Management of lipid abnormalities varied: most patients (68.8%) did not receive specific treatment, while others were managed with atorvastatin (12.5%), rosuvastatin (12.5%), or a combination of rosuvastatin 20 mg and evolocumab (6.3%). At the time of assessment, 62.5% continued to experience ongoing side effects. Overall, the data indicate a favorable response rate but a high incidence of lipid-related adverse events requiring ongoing monitoring (see Table 1).

**Table 1:** Demographic and clinical characteristics of patients (n = 16). Categorical data are presented as frequencies, while continuous data are presented as mean ± standard deviation (SD). DPCP: diphenylcyclopropenone; LDL: low-density lipoprotein

Variables	Description	Value
Associated diagnosis	Alopecia areata	15 (93.8%)
	Alopecia totalis	1 (6.3%)
Gender	Male	7 (43.8%)
	Female	9 (56.3%)
Age (years)	Mean ± SD	29.56 ± 9.21
Weight (kg)	Mean ± SD	67.12 ± 17.91
Previous treatment	Minoxidil 2.5 mg - tablet	1 (6.3%)
	Minoxidil 5% - lotion	8 (50.0%)
	Mometasone ointment	1 (6.3%)
	No	5 (31.3%)
	Topical and intralesional corticosteroid, DPCP, minoxidil, anthralin	1 (6.3%)
Starting dose	5 mg BID	12 (75.0%)
	5 mg daily	4 (25.0%)
Duration of treatment (year)	Mean ± SD	3.81 ± 1.27
treatment effectiveness	Ineffective	5 (31.3%)
	Effective	11 (68.8%)
Side effect	Hyperlipidemia	14 (87.5%)
	Hyperlipidemia, headache	1 (6.3%)
	No	1 (6.3%)
LDL (mmol/L)	Mean ± SD	3.07 ± 0.49
Management of side effect comment	Atorvastatin 20 mg	2 (12.5%)
	No	11 (68.8%)
	Resouvastatin 10 mg	2 (12.5%)
	Resouvastatin 20 mg, evolocumab	1 (6.3%)
Ongoing side effects	Yes	10 (62.5%)
	No	6 (37.5%)

The association between treatment response and various clinical characteristics among the 16 patients was evaluated. Overall, no significant relationships were identified between treatment effectiveness and most of the assessed variables, including diagnosis type (p = 0.126), gender (p = 0.838), age (p = 0.933), weight (p = 0.069), previous treatment history (p = 0.471), treatment duration (p = 0.978), LDL levels (p = 0.493), or the type of side effects experienced (p = 0.259). Although patients with AA demonstrated a higher proportion of effective responses (100%) compared with those with alopecia totalis, this difference did not reach statistical significance. Similarly, demographic characteristics such as age and gender showed no meaningful impact on treatment outcomes.

The only variable showing a statistically significant association with treatment response was the presence of ongoing side effects (p = 0.018). A higher proportion of patients in the effective group (81.8%) continued to experience side effects compared with the ineffective group (20.0%). This suggests that patients who responded positively to treatment either remained on therapy longer or required higher effective doses, resulting in more persistent adverse effects. Due to side effects, the treatment regimen was changed to other JAK inhibitors in six patients. Most other clinical characteristics were not significantly associated with treatment response. Taken together, while most clinical characteristics did not predict treatment success, the significant association with ongoing side effects highlights an important clinical consideration that warrants further investigation (Table 2).

**Table 2:** Association between the treatment response and clinical characteristics of patients. Categorical variables were analyzed using the chi-square test or Fisher’s exact test as appropriate. Continuous variables were analyzed using the independent samples t-test. Data are presented as numbers (%) or mean ± standard deviation (SD). A p-value < 0.05 was considered statistically significant. * shows that the p-value is significant at <0.05. LDL: low-density lipoprotein

Variables	Description	Ineffective (n = 5)	Effective (n = 11)	Test used	P-value
Associated diagnosis	Alopecia areata	4 (80.0%)	11 (100.0%)	Fisher’s exact test	0.126
	Alopecia totalis	1 (20.0%)	0 (0.0%)		
Gender	Male	2 (40.0%)	5 (45.5%)	Chi-square test	0.838
	Female	3 (60.0%)	6 (54.5%)		
Age (years)	Mean ± SD	29.20 ± 12.17	29.73 ± 8.24	Independent t-test	0.933
Weight (kg)	Mean ± SD	54.60 ± 15.10	72.81 ± 16.27	Independent t-test	0.069
Previous treatment	Minoxidil 2.5 mg (tablet)	1 (20.0%)	0 (0.0%)	Fisher’s exact test	0.471
	Minoxidil 5% (lotion)	3 (60.0%)	5 (45.5%)		
	Mometasone ointment	0 (0.0%)	1 (9.1%)		
	No prior treatment	1 (20.0%)	4 (36.4%)		
	Combination topical/intralesional therapies	0 (0.0%)	1 (9.1%)		
Duration of treatment (years)	Mean ± SD	3.80 ± 1.09	3.00 ± 1.40	Independent t-test	0.978
LDL (mmol/L)	Mean ± SD	3.24 ± 0.66	3.00 ± 0.42	Independent t-test	0.493
Side effects	Hyperlipidemia	4 (80.0%)	10 (90.9%)	Fisher’s exact test	0.259
	Hyperlipidemia + headache	1 (20.0%)	0 (0.0%)		
	None	0 (0.0%)	1 (9.1%)		
Changing to the new JAK inhibitor	Yes	1 (20.0%)	9 (81.8%)	Fisher’s exact test	0.018*
	No	4 (80.0%)	2 (18.2%)		

## Discussion

This study represents one of the longest real-world follow-ups of tofacitinib treatment for AA (six years) in a Saudi Arabian population, exceeding the duration of most international reports, which typically span only one to two years. For example, one previously published investigation assessed the efficacy and safety of tofacitinib over a two-year period (January 2016 to January 2018) [13], while another study evaluated patients treated for approximately one year (January to December 2018) [14]. In the Middle East, Al Ali et al. conducted the longest real-world follow-up to date, including 45 patients treated with tofacitinib for 12 to 72 months (mean 30 months), of whom 78% achieved moderate to significant hair regrowth. The treatment was generally well tolerated, and any hair shedding observed after discontinuation typically reversed once therapy was resumed [11]. Many patients in the present cohort achieved and sustained hair regrowth throughout the follow-up period, underscoring the potential of tofacitinib to provide long-term disease control, even within a relatively small sample. Comparable findings have been reported in previous studies. In an open-label pilot trial, Jabbari et al. observed significant hair regrowth within three to six months of treatment [15]. Another real-world follow-up study also demonstrated meaningful regrowth but highlighted the difficulty in maintaining a long-term response over extended periods [16]. Collectively, these studies point to consistent therapeutic benefits of tofacitinib, while the current six-year follow-up uniquely provides evidence of prolonged disease control and treatment durability beyond what has been previously documented. Pediatric patients in this cohort demonstrated favorable treatment responses, providing valuable long-term real-world evidence in a population with limited data on tofacitinib use.

Existing literature is largely restricted to small case series and isolated reports with short follow-up. For example, a study of four pediatric patients showed short-term improvement [17], and a recent case report described early response in a six-year-old child with severe AA [18]. Unlike these reports, our multi-year cohort offers insight into longer-term outcomes, helping address an important evidence gap regarding sustained efficacy and safety in pediatric patients. Several patients in the current study required escalated doses of 15-20 mg/day higher than the dosing regimens commonly reported in clinical trials - yet these doses were generally well tolerated. Evidence from the literature supports the safety and effectiveness of such higher doses in real-world practice. In a case series of 10 pediatric patients with AA, nine received 20 mg/day of tofacitinib (some titrated from 10 mg/day and others started directly at 20 mg/day), and all achieved complete hair regrowth within one to six months; no adverse events or laboratory abnormalities were observed except mild acne in one patient [19]. Similarly, a study involving 13 adult patients treated with oral tofacitinib reported that five individuals received 20 mg/day and one received 15 mg/day. Together, these findings strengthen the evidence that escalated dosing may be both effective and safe in selected patients, supporting reconsideration of dose-response strategies in real-world AA management [5].

Elevated LDL cholesterol levels observed in several patients are consistent with the well-recognized metabolic effects of tofacitinib. Tofacitinib has been shown to cause modest, dose-dependent increases in LDL cholesterol (LDL-C) and high-density lipoprotein cholesterol (HDL-C) during the early weeks of therapy, which typically plateau with continued treatment and rarely necessitate discontinuation when appropriately monitored. In our cohort, tofacitinib-associated lipid abnormalities were managed pharmacologically, including statin initiation when indicated, without treatment discontinuation. This approach is supported by a retrospective AA study by Ibrahim et al., in which dose-dependent elevations in LDL-C and other lipids were successfully managed through dose adjustment or statin therapy [5]. Although no AA-specific studies have systematically evaluated statin use for tofacitinib-associated dyslipidemia, evidence from rheumatoid arthritis populations demonstrates that atorvastatin effectively reverses LDL-C elevations induced by tofacitinib, supporting the feasibility of maintaining therapy with appropriate metabolic monitoring and timely intervention [20]. Although PCSK9 inhibitors are generally reserved for patients at high cardiovascular risk, evolocumab was initiated in this case due to refractory LDL-C elevation despite statin therapy. No cardiovascular events were observed during follow-up, and lipid abnormalities were managed pharmacologically without discontinuation of tofacitinib. Six patients (37.5%) of the total cohort required treatment switching to baricitinib during follow-up. In four patients, treatment switching was initially prompted by suboptimal or absent clinical response; however, tofacitinib was continued until the development of hyperlipidemia, which ultimately became the primary reason for switching therapy. In the remaining two patients, an effective clinical response had been achieved, but treatment was also discontinued and switched because of hyperlipidemia. The availability of baricitinib at our center allowed for this change to optimize long-term safety while maintaining disease control [21].

In parallel, the availability of ritlecitinib has expanded therapeutic options for moderate-to-severe AA. Phase 2b/3 and phase 3 trials have shown that ritlecitinib significantly improves hair regrowth, quality of life, and psychological well-being, including in adolescent populations [22]. These developments highlight the need for future comparative studies to better define the role of tofacitinib among JAK inhibitors.

There are several limitations to this study that should be considered. The small sample size limits the statistical power of the analysis and reduces the generalizability of the findings to broader patient populations. In addition, the retrospective design of the study relies on the accuracy and completeness of electronic medical records, which may expose the results to missing data or information bias. Furthermore, tofacitinib is not approved by the U.S. Food and Drug Administration (FDA) for the treatment of AA, and its off-label use may limit the applicability of the findings to routine clinical practice. Conducting the study at a single center may have introduced selection bias, and the absence of a control or comparison group limits the ability to draw causal inferences or compare effectiveness with other treatment options.

## Conclusions

This study represents one of the longest real-world evaluations of oral tofacitinib for AA in a Saudi population, with follow-up of up to six years. Tofacitinib achieved sustained clinical improvement in a substantial proportion of patients with moderate-to-severe disease, with nearly two-thirds attaining an effective response. Treatment was generally well tolerated; lipid abnormalities were common but manageable, and no serious adverse or cardiovascular events were observed. Most baseline characteristics did not predict response, highlighting disease heterogeneity. Overall, these findings support the feasibility of long-term tofacitinib therapy in carefully selected patients, with structured monitoring and continued real-world evaluation.
